# Increased Engraftment of Human Short Term Repopulating Hematopoietic Cells in NOD/SCID/IL2rγ^null^ Mice by Lentiviral Expression of NUP98-HOXA10HD

**DOI:** 10.1371/journal.pone.0147059

**Published:** 2016-01-13

**Authors:** Allistair Abraham, Yoon-Sang Kim, Huifen Zhao, Keith Humphries, Derek A. Persons

**Affiliations:** 1 Division of Experimental Hematology, St Jude Children's Research Hospital, Memphis, Tennessee, United States of America; 2 Terry Fox Laboratory, British Columbia Cancer Agency, Vancouver, British Columbia, V5Z 1L3, Canada; Wake Forest Institute for Regenerative Medicine, UNITED STATES

## Abstract

Techniques to expand human hematopoietic stem cells ex-vivo could be beneficial to the fields of clinical hematopoietic stem cell transplantation and gene therapy targeted at hematopoietic stem cells. NUP98-HOXA10HD is a relatively newly discovered fusion gene that in mouse transplant experiments has been shown to increase numbers of hematopoietic stem cells. We evaluated whether this fusion gene could be used to expand engrafting human primitive CD34+ cells in an immunodeficient mouse model. Gene transfer was achieved using a lentiviral based vector. The engraftment of mobilized peripheral blood human CD34+ cells grown in culture for one week after gene transfer was evaluated 3–4 months after transplant and found to be 2–3 fold higher in the NUP98-HOXA10HD groups as compared to controls. These data suggest an expansive effect at least at the short term human repopulating cell level. Further evaluation in long term repopulating models and investment in a NUP98-HOXA10HD protein seems worthy of consideration. Additionally, the results here provide strong impetus to utilize NUP98-HOXA10HD as a tool to search for underlying genes and pathways involved in hematopoietic stem cell expansion that can be enhanced and have an even more potent expansive effect.

## Introduction

The identification of a true human hematopoietic stem cell (HSC) defined as one that indefinitely self-renews and is capable of repopulating the entire hematopoietic system remains elusive. Manipulating hematopoietic grafts using cell surface markers (e.g. CD34+ or CD133+ positive selection) can enrich the number of HSCs in a sample. Using the example of peripherally mobilized blood stem cell grafts a threshold of CD34+ content per body weight of the recipient can be used to predict the likelihood of engraftment after clinical transplantation[1]. It still remains unknown exactly which of these CD34+ cells are the ones responsible for life long hematopoiesis. Finite numbers of HSCs in hematopoietic grafts used for clinical transplantation can limit their use if there are insufficient total cell numbers relative to the body size of the transplant recipient. The ability to achieve durable engraftment of HSCs that have undergone gene transfer to correct genetic disorders is also dictated by HSC number as is successful engraftment with use of submyeloablative conditioning to avoid transplant related morbidity. Thus efforts to improve both scenarios have focused on methods to expand and maintain HSCs from a functional point of view. Earlier approaches to ex-vivo expansion have employed optimization of liquid culture conditions, using cytokines shown to affect hematopoietic progenitor cell proliferation and differentiation such as erythropoietin, granulocyte colony stimulating factor, stem cell factor, thrombopoietin, FLt-3 ligand, interleukin-3 (IL-3) and IL-6[2]. One such method optimized for CD34+ umbilical cord blood cells showed an increase in progenitor expansion as demonstrated by increased colony formation in progenitor assays[3]. Subsequent experiments in a fetal sheep transplant model using human CD34+ cord blood cells expanded using the same method showed a more rapid engraftment but lacked long term engraftment and cells could not be serially transplanted[4]. This observation has raised concern over expansion methods negatively affecting the more primitive long term progenitors and HSCs and in clinical trials both an expanded and unexpanded cord blood product are concomitantly infused[5]. More recently, work using newer expansion techniques including small molecules (Notch ligand, StemRegenin 1, Um171), other culture conditions (copper chelation, nicotinamide, MSC co-culture) and cell modification (PGE-2, fucosylation) have shown expansive effects on umbilical cord blood cells[6–13]. Consistent long term repopulation data in human cells has not yet been reported but there are a number of Phase I/II trials that have been completed with Phase II/III studies planned[14].

Hematopoietic cell development has been shown to be influenced by Homeobox (HOX) genes and overexpression of these genes, such as in the case of HOXB4, can increase the number of HSCs[15–17]. HOXB4 overexpression by retroviral vector in adult mouse bone marrow cells resulted in a 40 -fold net expansion of HSCs in short term (7–10 day) in vitro culture as evidenced by limiting dilution transplantation experiments[16]. In experiments using co-culture with HOXB4 protein secreting cells with human cord blood CD34+ cells, transplantation into NOD-SCID mice showed a 2.5 fold increase in repopulating cells; a modest expansion compared to that seen with mouse HSCs[15]. Furthermore, in non-human primate models while showing improved short term engraftment of HOXB4 overexpressing cells, long term engraftment levels were disappointingly lower with granulocytic marking being 20% and less[18]. Concern has been raised about HOXB4 overexpression perturbing hematopoiesis with decreased B lymphocyte output and decreased myeloid and erythroid progenitors[19].

In pursuit of a protein that may have more effective expansion on human HSCs and avoid untoward effects on hematopoiesis, further investigation into other HOX gene effects has been undertaken. The transcriptional co-activator Nucleoporin98 has been naturally found fused with certain HOX genes and similar HSC expansive effects as seen with HOXB4 have been observed in the mouse model[20]. Notably the fusion gene Nucleoporin98-HOXA10 (NUP98-HOXA10) has shown HSC expansion effects superior to HOXB4 and Nucleoporin98-HOXB4. In murine transplant experiments, NUP98-HOXA10 expression was achieved in 5-fluorouracil treated wild type bone marrow cells that were transduced with a retroviral vector, and transplanted by limiting dilution into lethally irradiated recipients. Compared to controls, the NUP98-HOXA10 group had an over 1000-fold expansion of HSCs[20]. The Nucleoporin98-HOXA10 fusion has been further engineered such that only the homeodomain portion of HOXA10 is fused to N-terminus of Nucleoporin98 (NUP98-HOXA10HD) with the intention to maintain its expansive effects on HSCs while minimizing the potential to disturb normal hematopoiesis [20, 21]. Using the NUP98-HOXA10HD fusion, HSC expansion was found to be comparable to NUP98-HOXA10 and in fact found that if the cells were cultured for three weeks prior to transplant, a 10,000-fold expansion was observed. Importantly there was no negative impact seen on hematopoiesis with equal contribution to all lineages and mice followed even over a year post transplant had no evidence of myelodysplasia[20]. Based on these encouraging findings we therefore set out to evaluate if enforced expression of the NUP98-HOXA10HD gene delivered by lentiviral vector transfer could have similar effects on human mobilized peripheral blood CD34+ cells as those seen in murine marrow transplant experiments. Positive results would support the use of NUP98-HOXA10HD as a powerful tool to identify critical genes/pathways involved in HSC self-renewal and as a means to sustain or improve the HSC content in grafts maintained in culture and potentially improve the transfer efficiency in gene therapy where HSCs are the main target.

## Results

### Lentiviral vector production

**Fig 1:**
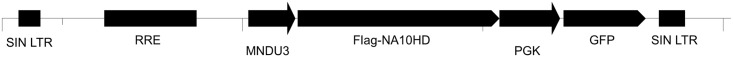
NUP98-HOXA10HD (NA10HD) lentiviral vector construct. Self inactivating lentiviral vector encoding (SIN LTR), *rev* responsive element (RRE), modified MLV long terminal repeat promoter (MNDU3), FLAG tagged NUP98-HOXA10HD (FLAG-NA10HD), mouse phosphoglycerate kinase promoter (PGK), enhanced green fluorescent protein (GFP).

**Fig 2:**
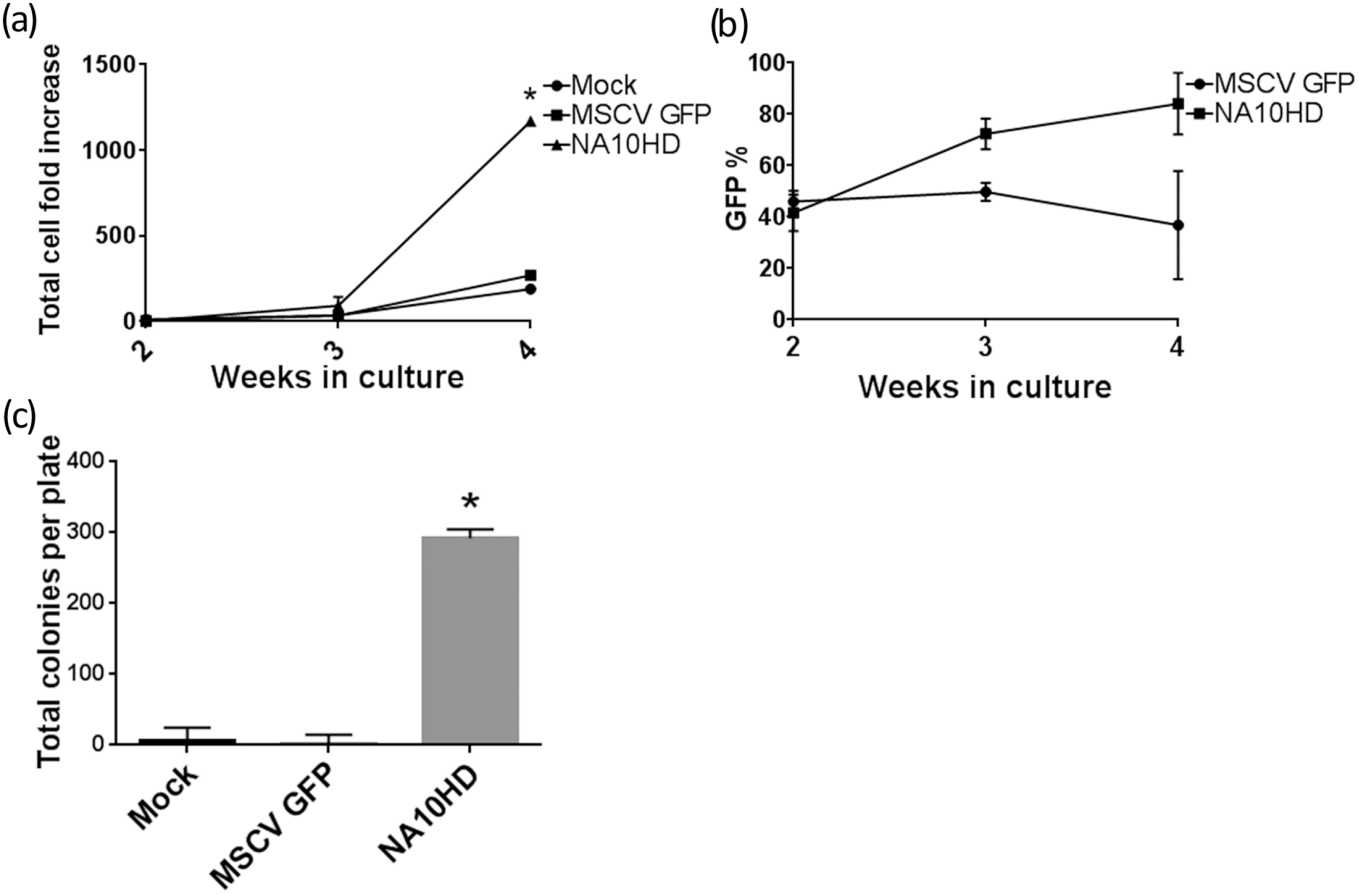
Biologic activity of NUP98-HOXA10HD in mouse bone marrow. (a) Growth curve of lineage depleted C57BL/6 mouse bone marrow cells transduced with GFP control or NUP98-HOXA10HD-GFP vector (NA10HD) as compared to untransduced mock cells grown in liquid culture, n = 2, at 4 weeks NA10HD has more expansion vs. Mock and MSCVGFP (p = 0.013 and p = 0.024 respectively, paired 2-tailed t-test, n = 2), (b) Percentage of GFP positive cells in liquid culture over time showing an enrichment of GFP positive cells in the NA10HD group over time, (c) CFU-C assay of mouse marrow cells that had been in culture for 39 days and plated at 6000 cells/plate, NA10HD has more colonies vs. Mock and MSCVGFP (p<0.0001, unpaired 2-tailed t-test, n = 2).

The lentiviral vector used in these experiments containing the NUP98-HOXA10HD fusion gene (Fig 1) had a transducing titer of 2–3 x 10^7^ units/ml as measured on 293T cells. Biological activity of the fusion gene was first confirmed in an *in vitro* mouse marrow model (Fig 2a) where after about three weeks in culture the NUP98-HOXA10HD groups showed a growth advantage (p = 0.013 and 0.024 respectively). There was also a concomitant increase in the percentage of green fluorescent protein (GFP) positive cells seen in the NUP98-HOXA10HD group over time (Fig 2b). After extensive culture for 39 days, the NUP98-HOXA10HD group had a large number of CFUs whereas control GFP and Mock groups had little measurable CFUs remaining (p<0.0001, Fig 2c). NUP98-HOXA10HD transduced cells also showed the ability to renew in re-plating assays (data not shown).

### Transduction of Human CD34+ cells

**Fig 3:**
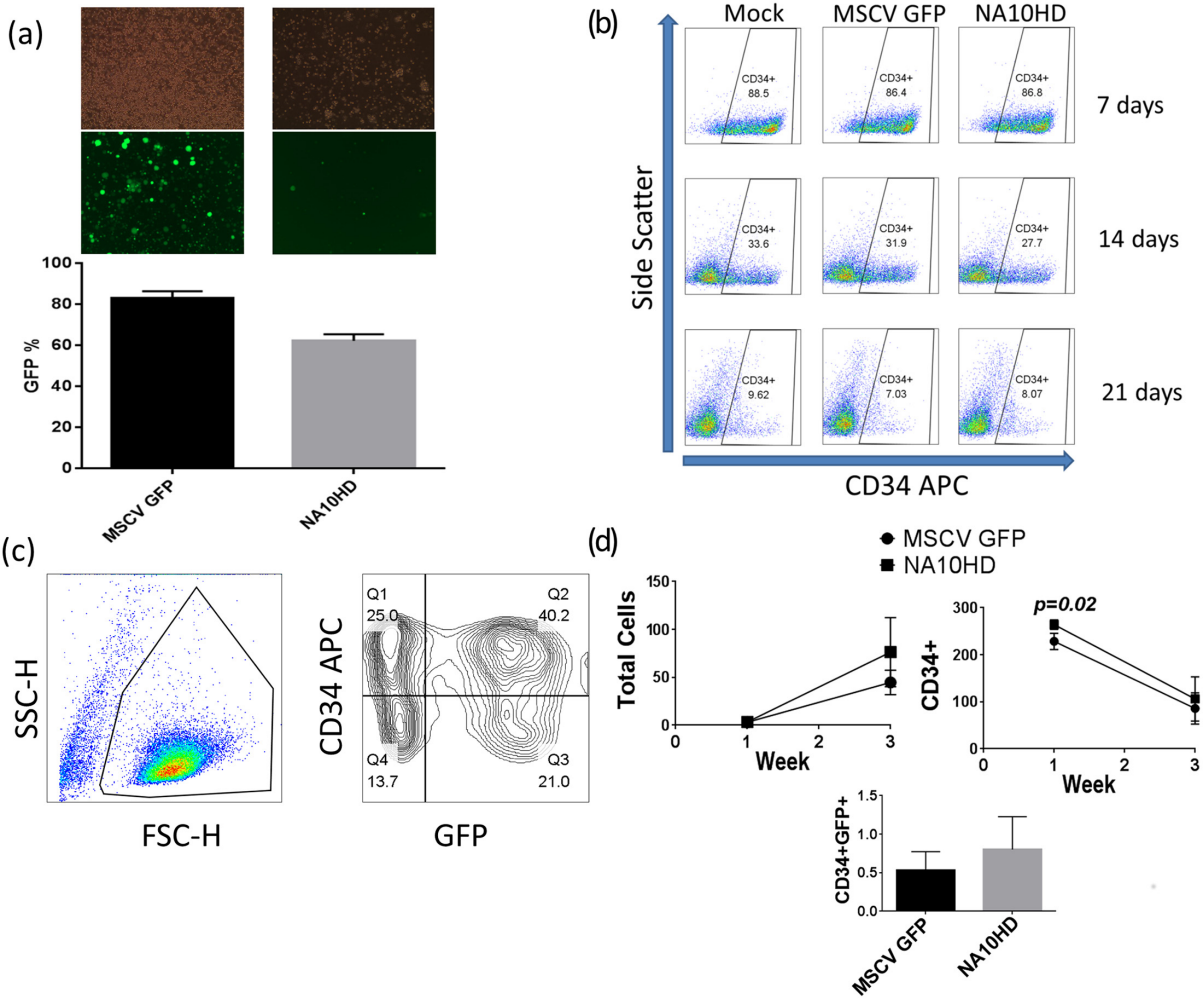
Transduction and culturing of human peripheral blood CD34+ cells. (a) GFP expression of CD34+ cells 1 week post lentiviral transduction as measured by brightfield (top row) and fluorescent (middle row) microscopy and flow cytometry (bottom row) 1 week post lentiviral vector exposure—vCL20c MSCV GFP as GFP control (MSCV GFP), vCCL-c-MNDU3 FLAG-NA10HD PGK GFP (NA10HD), (b) Similar appearance of CD34+ cells in culture at 1, 2 and 3 weeks post-transduction amongst the untransduced (Mock), MSCV GFP and NA10HD groups, (c) Gating strategy fog assessment of GFP positive CD34+ cells in long-term cell culture, (d) Cumulative fold increase analysis for total cells, CD34+ and CD34+GFP+ in culture for 3 weeks shows significantly higher CD34+ expansion at 1 week post lentiviral transduction in the NA10HD group (264 vs 228, p = 0.02, paired t-test, n = 5). Total cell, CD34+ and CD34+GFP+ cell expansion in NA10HD vs. MSCVGFP at 3 weeks of cell culture was higher but did not meet statistical significance (p = 0.267, 0.344 and 0.323 respectively, paired t-test, n = 5).

**Fig 4:**
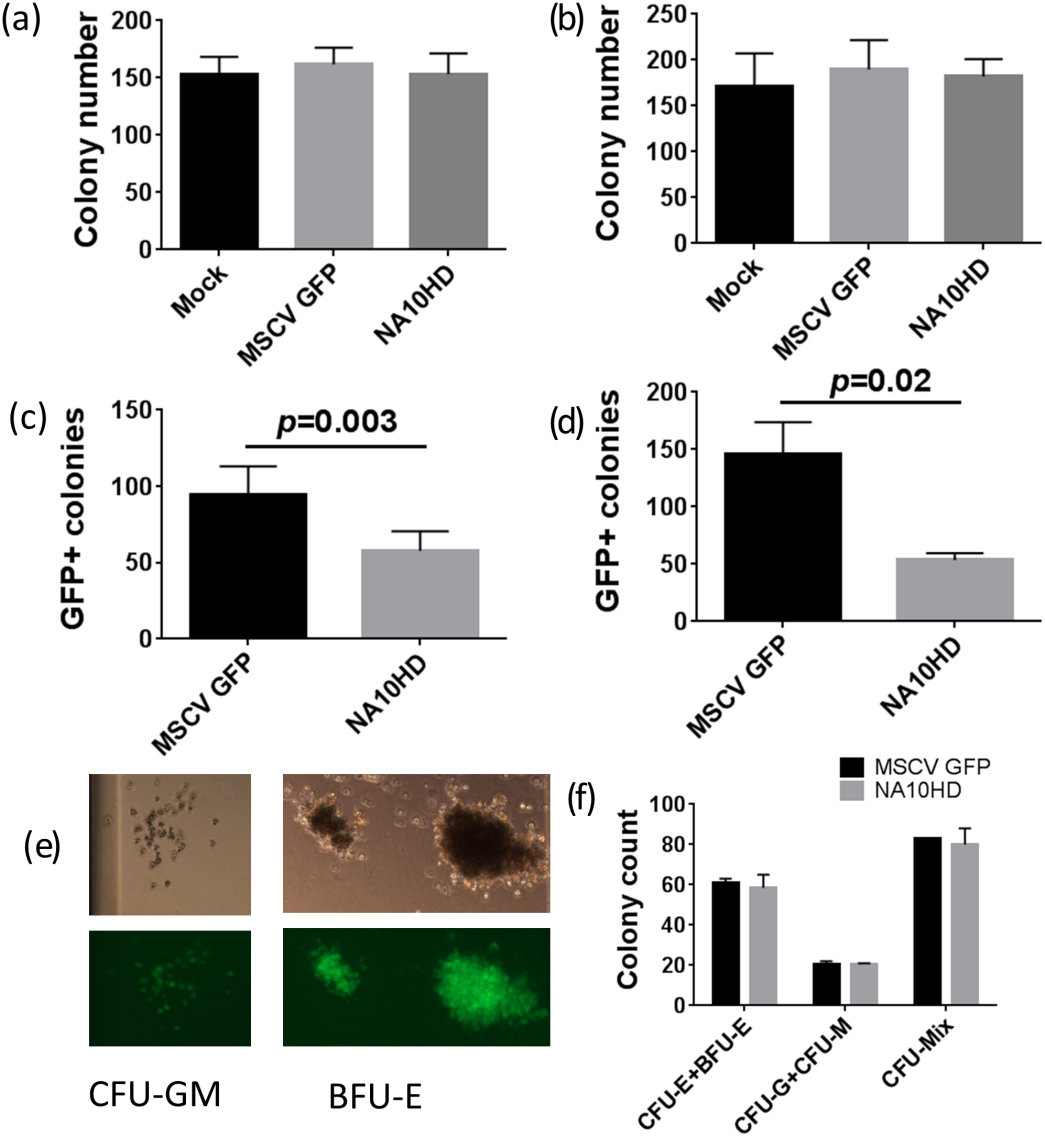
CFU-C assay of human CD34+ cells from four donors transduced with control GFP vector or NA10HD. (a) and (b) showing similar total colony numbers from CD34+ cells in culture 1 day and 10–14 days post transduction, (n = 7), (c) and (d) show the GFP positive colony numbers are lower in the NA10HD group compared to MSCVGFP for cells in culture 1 day and 10–14 days post transduction, (p = 0.003 and 0.02 respectively, paired t-test, n = 7), (e) Colonies (CFU-GM and BFU-E) obtained after NA10HD transduction, (f) Similar number of erythroid colonies between MSCVGFP and NA10HD groups suggesting that erythroid differentiation is not blocked by NA10HD expression (n = 2).

The mean gene transfer efficiencies of the GFP control vector (MSCVGFP) and NA10HD vector in human CD34+ cells as measured by GFP positivity in the first week post transduction in invitro experiments was 82.4% and 62.8% respectively (Fig 3a). The morphological characteristics of CD34+ cells by flow cytometry in the NA10HD group was similar post-transduction to the MSCV GFP control and untransduced Mock groups (Fig 3b). At 1 week post transduction there was a statistically higher fold increase in CD34+ cells in the NA10HD group compared to MSCVGFP (264 vs. 228, p = 0.02), and at 3 weeks the total cell, CD34+ and CD34+GFP+ fold increase was higher in the NA10HD group but this was not statistically significant (Fig 3c and 3d). CFU-C assay of the cells 1 day and 10–14 days after transduction did not show a significant difference in the total number of colonies amongst the groups (Fig 4a and 4b) but interestingly had lower GFP positive colonies in the NA10HD group than in the MSCVGFP group (p = 0.003 and 0.02 respectively, Fig 4c and 4d). The distribution of colony types including erythroid colonies was similar between the transduced groups (Fig 4e and 4f).

### Engraftment of transduced human CD34+ cells in NSG mice

**Fig 5:**
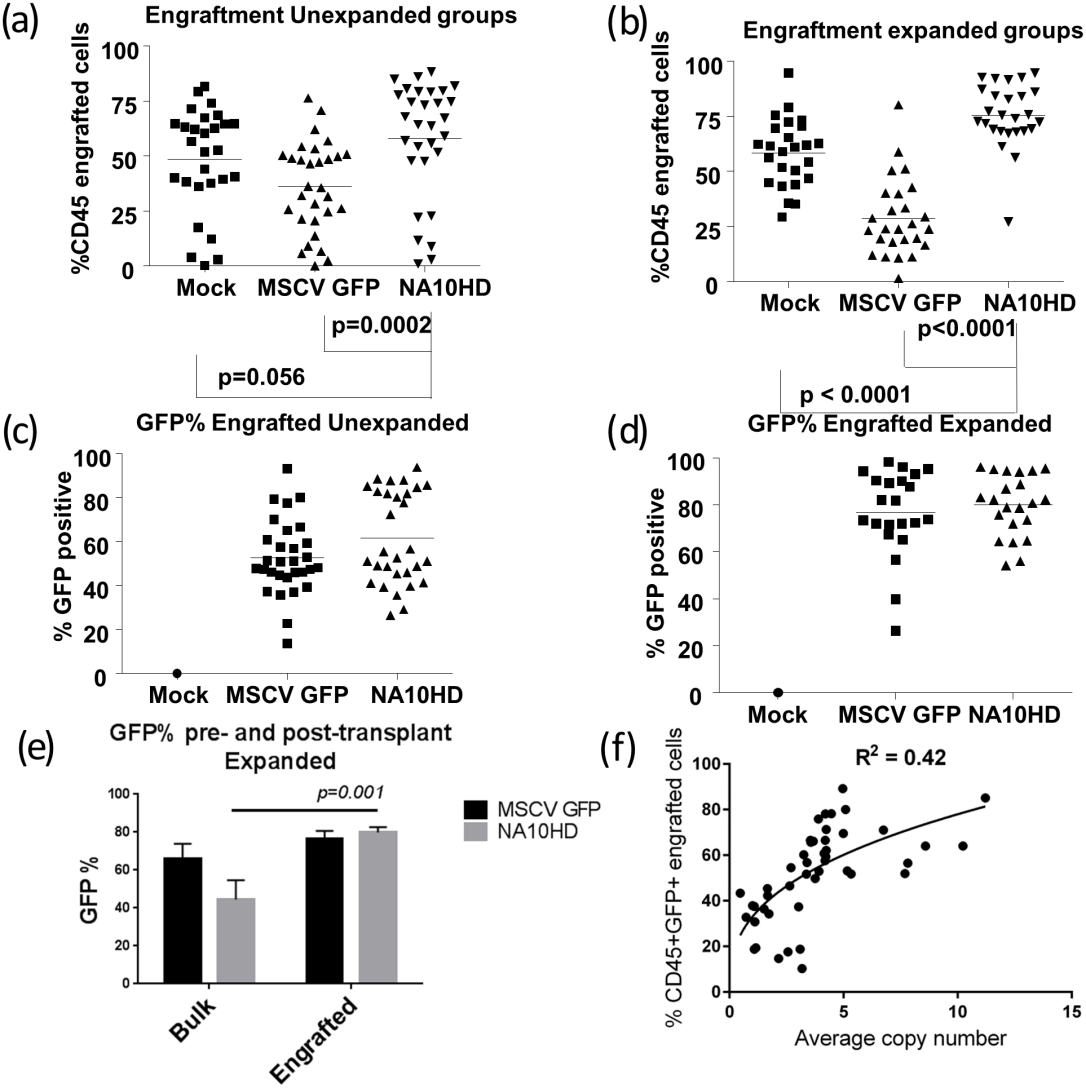
NUP98-HOXA10HD results in increased engraftment of CD45+ cells (a) and (b)–Human nucleated hematopoietic cell engraftment (CD45+) as percentage of total bone marrow harvested from NSG mice 12–15 week post transplantation with lentiviral transduced CD34+ cells. (a) represents transplantation 24 hours post transduction, (b) represents cells “expanded” in culture 1 week post transduction prior to transplant both showing NA10HD groups with significantly higher engraftment that MSCVGFP control, (c) and (d)–corresponding GFP expression of CD45+ cells. (e) Change in GFP% from input CD34+ bulk cells to engrafted CD45+ cells for “expanded” groups showing a significant increase in the NA10HD but not MSCV GFP group (p = 0.001 and 0.29 respectively, 2-tailed upaired t-test), (f) Average vector copy number per cell analysis of NA10HD vector as determined by qPCR shows a weakly positive correlation with engrafting transduced cells (CD45+GFP+) up to a copy number of approximately 5 and then plateaus, suggesting that the expansive effect of NA10HD increases with higher expression per engrafted cell but the effect has a ceiling. For (a)-(d), a p-value of 0.05 was considered significant, comparisons were performed using Mann-Whitney U test.

**Fig 6:**
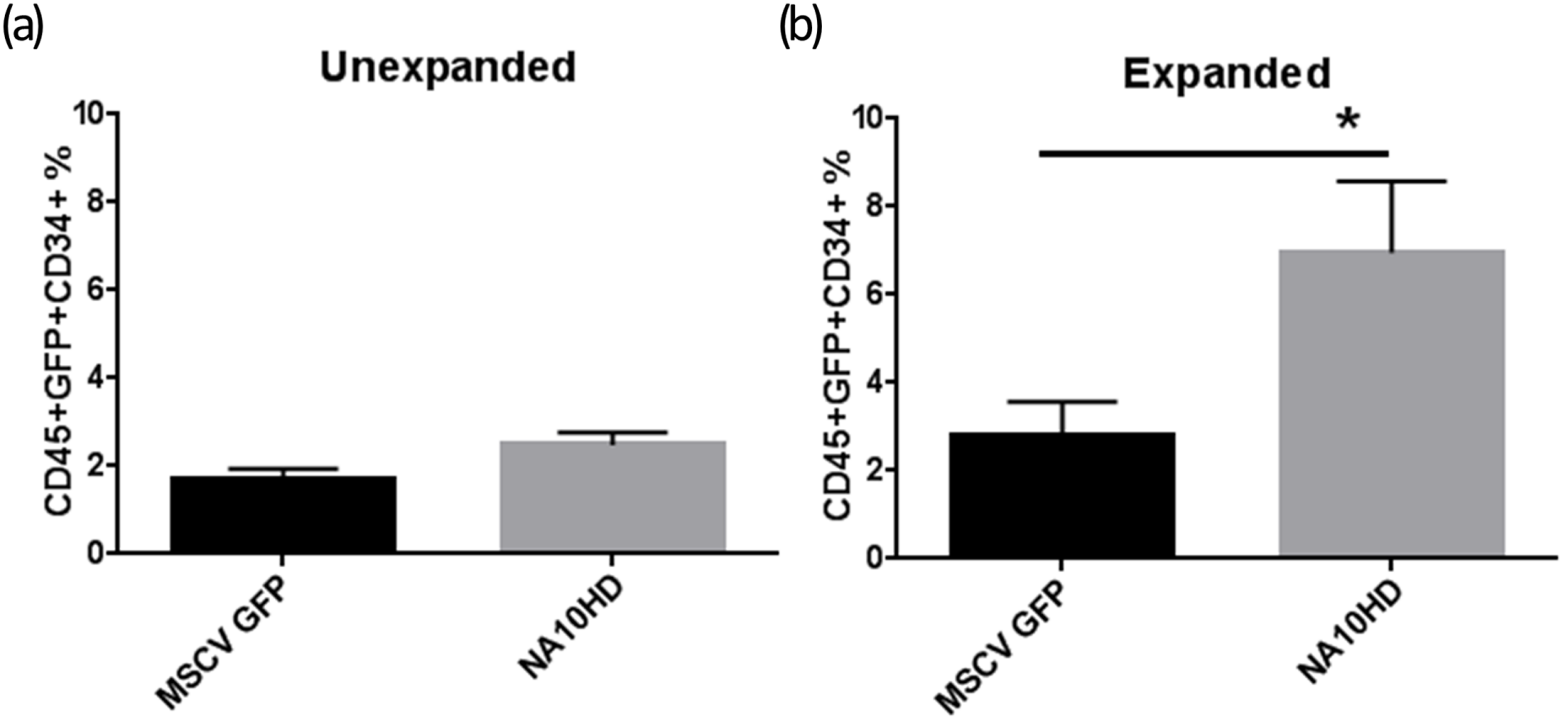
Increased engraftment of CD34+GFP+ cells with NA10HD. CD45+CD34+GFP+ cells recovered from bone marrow harvested from NSG mice 12–15 week post transplantation with lentiviral transduced CD34+ cells that remained in culture for 24 hours (“Unexpanded”) after transduction (a) or expanded for 1 week (“Expanded”) post-transduction prior to transplant (b). Statistical comparisons performed using Mann-Whitney U testing with a p-value of 0.05 being considered significant. In the unexpanded group there was a higher percentage of CD45+CD34+GFP+ cells in the NA10HD group compared to MSCV GFP that trended toward but did not meet statistical significance (2.4 vs 1.7%, p = 0.06, n = 30 mice per condition), the Expanded group showed a higher CD45+CD34+GFP+ percentage for NA10HD (6.96 vs 2.8%, p = 0.006, n = 26 mice per condition).

**Fig 7:**
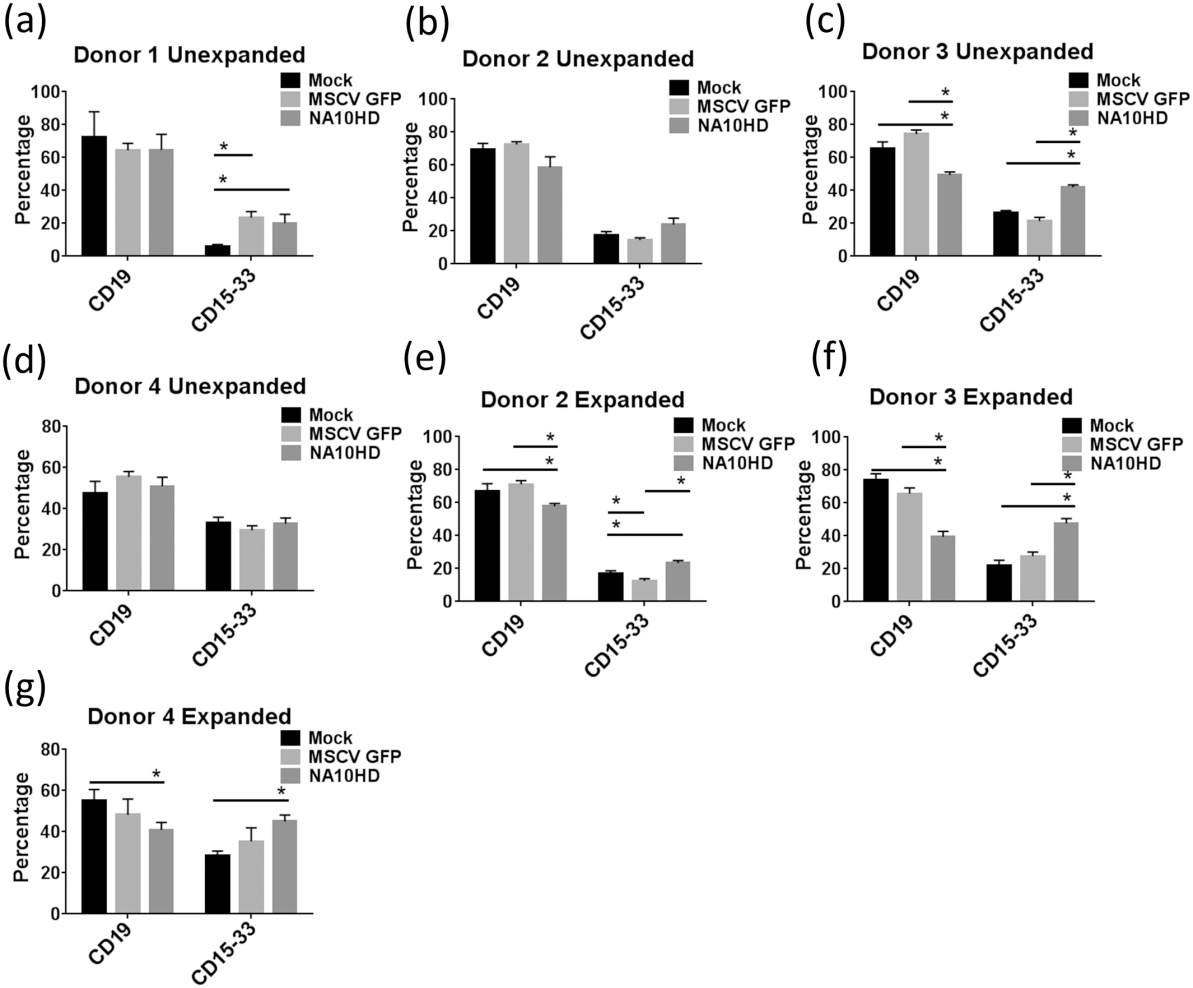
Distribution of CD19+ and CD15/33+ cells amongst CD45+ engrafted cells. CD45+ cells recovered from bone marrow harvested from NSG mice 12–15 week post transplantation with lentiviral transduced CD34+ cells that remained in culture for 24 hours after transduction (a)-(d) or for one week after transduction (Expanded) (e)-(g). Statistical comparisons performed using Mann-Whitney U testing with a p-value of 0.05 being considered significant (*).

In order to establish whether NUP98-HOXA10HD could expand primitive human repopulating cells we transduced mobilized peripheral blood CD34+ cells from four different donors with the NUP98-HOXA10HD lentiviral vector followed by liquid culture of either 24 hours or extended culture of one week, and then transplanted the cells into NOD/SCID/IL2rγ^null^ mice (NSG). The mean gene transfer efficiencies of the bulk human CD34+ graft using the NUP98-HOXA10HD vector was 56.6% and 44.5% at days 4 and 11 post transduction respectively. The corresponding MSCV GFP control values were 73.1% and 66.2%. Engraftment was measured as the percentage of human CD45+ cells in the NSG mouse marrow at the time of analysis 3–4 months post-transplant. Taken as a composite, in the groups where cells were transplanted into mice without extended culture (Unexpanded), NUP98-HOXA10HD showed a trend toward higher engraftment compared to the untransduced Mock group (p = 0.056) and a statistically significant higher engraftment compared to the control GFP transduced group (p = 0.0002) (Fig 5a). In the extended culture groups the engraftment in NUP98-HOXA10HD groups was significantly higher compared to Mock and GFP controls (p = <0.001 in both cases) (Fig 5b). There were insufficient cells to carry out the extended culture condition arm for Donor 1. There appeared to be two populations of mice based on percentage of GFP+ engrafting cells and this was more evident in the Unexpanded groups. However no clear pattern was observed between level of GFP expression and CD45+ cell engraftment and a weak positive correlation was found between the two (Spearman correlation, *r* = 0.519, p<0.001; S1 Fig). The GFP positive percentage in the NUP98-HOXA10HD group greatly increased from the time of transplant to the engraftment analysis as compared to the GFP control in the expanded group (p = 0.001, Fig 5e). The average vector copy number was 3.84 in the CD45+ engrafting cells and a weak positive logarithmic relationship (R^2^ = 0.4) was observed between copy number and percentage of transduced engrafting cells i.e. CD45+GFP+ (Fig 5f). This suggests increased engraftment with increased NUP98-HOXA10HD expression but this effect plateaued and was not consistent. In the unexpanded group there was a higher percentage of CD45+CD34+GFP+ cells in the NA10HD group compared to MSCV GFP that trended toward but did not meet statistical significance (2.4 vs 1.7%, p = 0.06, n = 30 mice per condition) and the Expanded group showed a higher CD45+CD34+GFP+ percentage for NA10HD (6.96 vs 2.8%, p = 0.006, n = 26 mice per condition, Fig 6). In subset analysis, the engrafted cells generally had a comparable lymphoid/myeloid distribution between NUP98-HOXA10HD and control groups and a small decrease in lymphoid output was observed, but not consistently (Fig 7).

### CFU-C assay of sorted CD45+ from NSG mice

**Fig 8:**
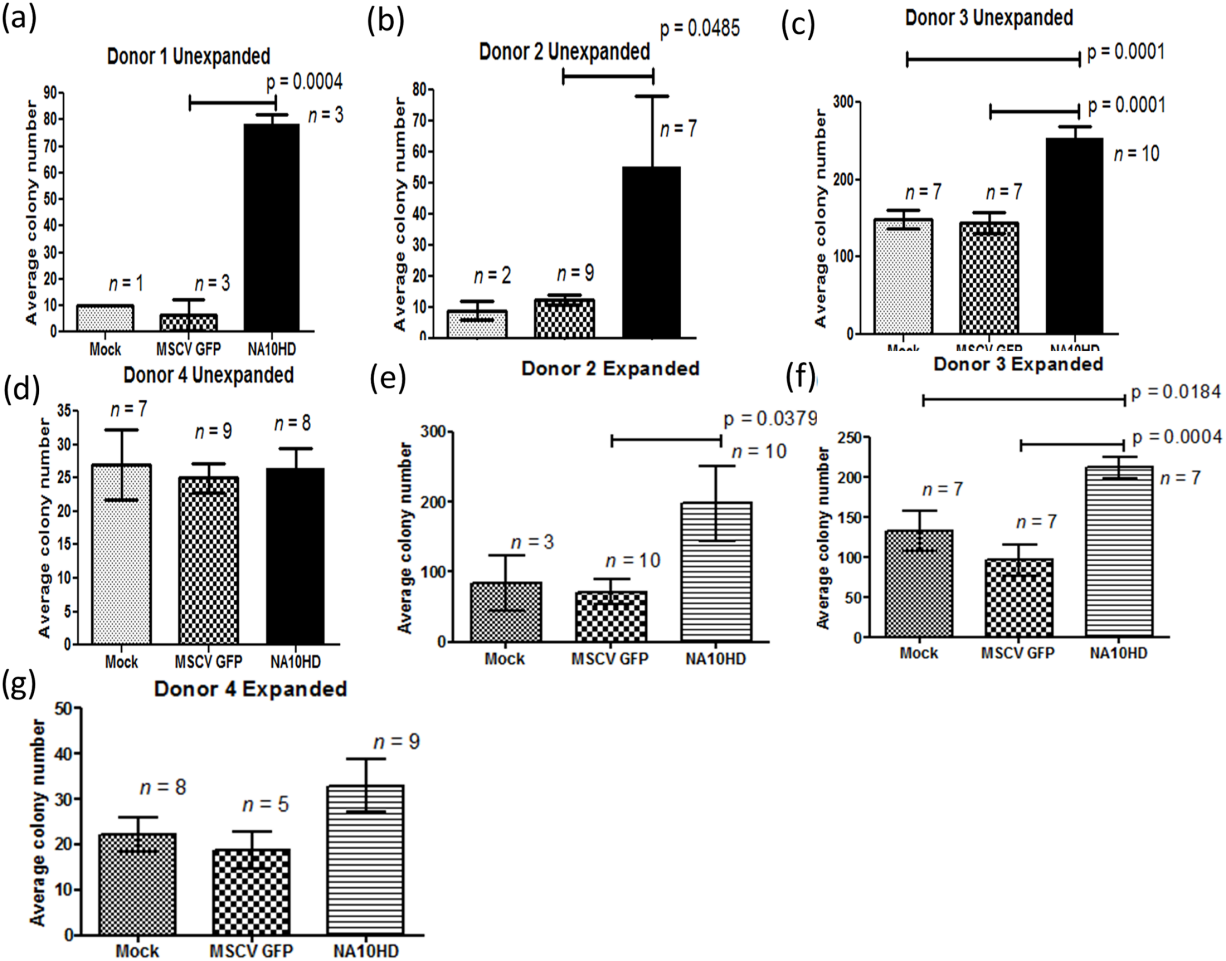
CFU-C assay of CD45+ engrafted cells recovered from bone marrow of NSG mice 12–15 week post transplantation with lentiviral transduced CD34+ cells. (a)–(d) Average colony numbers from four different human donors transplanted without expansion after transduction process. Donor 4 samples were inadvertently plated at one-third the intended number of cells per plate. (e)-(g) Average colony numbers from three different human donors transplanted after expansion in liquid culture for one week. Donor 4 samples were inadvertently plated at one-third the intended number of cells per plate. Donor 1 did not have sufficient CD34+ cells to perform the expanded cell experiments. Statistical comparisons performed using two-tailed upaired t-test with a p-value of 0.05 being considered significant.

If primitive repopulating cells had expanded one might predict that this would yield an increase in the frequency of colony forming cells (CFU). We therefore set up CFU-C assays by plating CD45+ cells sorted from the marrow of NSG mice after engraftment analysis was completed. The average colony number in the NUP98-HOXA10HD group was higher in the unexpanded arms in three out of the four donors (Fig 8a, 8b and 8c). In the expanded groups NUP98-HOXA10HD had higher CFU output for three donors, two of which were statistically significant (Fig 8e and 8f). One donor (Donor 4) had higher number of colonies in the NUP98-HOXA10HD group that approached but did not meet statistical significance (Fig 8g). Donor 3 NUP98-HOXA10HD showed a higher number of CFUs compared to both untransduced Mock and GFP controls at statistical significance for unexpanded (Fig 8c) and expanded (Fig 8f) conditions.

## Discussion

Ex vivo human HSC expansion has been the focus of many groups for over three decades now and remains an ongoing challenge. The field of hematopoietic stem cell transplantation could benefit from an efficient HSC expansion process which could decrease instances where a graft is deemed not suitable due to insufficient cells per body weight of the recipient. This has been a significant issue specifically with umbilical cord blood grafts being used for larger children and adults. Cell dose becomes even more of a problem with greater degree of HLA mismatch between graft and recipient, where it is seen that even higher doses of cells are needed in the mismatch scenario to minimize risk of non-engraftment. The field of gene therapy for hematologic disease remains plagued by less than desirable sustained gene modified cell efficiency. One could foresee that HSC expansion may improve the process by providing more starting material for gene transfer and further expansion of modified HSCs for transplant. HSC expansion efforts have initially focused on providing the right environment and culture conditions. Various cytokines have been shown to have effects on hematopoiesis that may promote HSC expansion including stem cell factor (SCF), granulocyte colony stimulating factor (G-CSF), thrombopoietin (TPO), IL-3, and IL-6[2]. While promising results have been obtained in mouse marrow transplant experiments, the expansive effects on human primitive cells have been modest. Further efforts have combined optimizing culture conditions with a supportive stromal cell layer in a co-culture system as well as a controlled environment with respect to oxygen tension, pH, and temperature all in attempts to simulate a bone marrow niche conducive to HSC expansion [5]. To date these efforts have not proven to efficiently expand human HSCs in a long term repopulation model. Small molecule compounds have also been screened for expansive properties and recently StemRegenin 1 (SR1) was shown to increase the engraftment of CD34+ umbilical cord blood cells in an immunodeficient mouse model by 17-fold [7]. Another small molecule, UM171, when used in combination with SR1 further enhances the formation of multilineage colony forming cells[22]. These promising results remain to be confirmed in a long term repopulation model. Additionally, it should be noted that the majority of published data on human HSC expansion has used umbilical cord cells as opposed to peripheral blood stem cells which have been more challenging to expand. This is of considerable importance for autologous gene therapy where patients are unlikely to have their umbilical cord blood stored but could provide peripheral blood stem cells for gene therapy.

With the continued search for an effective method to expand HSCs, transcription factors discovered to control hematopoiesis have also garnered attention. The Homeobox (Hox) family of genes play important roles not only in embryonic development but also in hematopoiesis[17]. Out of research involving effects of dysregulated Hox gene expression on hematopoiesis, HOXB4 was identified as a candidate to expand HSCs and overexpression experiments in mouse experiments showed that a 1000-fold expansion over controls could be achieved[16]. In moving to human cells the effects were substantially less with a 2–3 fold expansion in a short term repopulation model and concerns had been raised over disturbance of normal hematopoiesis leading to decreased lymphocyte output and decreased myeloid/erythroid progenitors[15, 19].

Fusions of Nucleoporin 98 with Hox genes (NUP98-*Hox* fusions) have been reported in cases of myeloid leukemias in patients[23]. NUP98-HOXA10 is a novel fusion designed to investigate the leukemogenic effect in a mouse model, and in these experiments the ability to block bone marrow differentiation was demonstrated[24]. In order to utilize such a gene for HSC expansion while allowing differentiation NUP98-HOXA10HD was created by a modification of the original fusion where only the N-terminus of Nuceloporin98 is joined to the homeodomain of HOXA10[20]. The resultant gene appears to have less likelihood of lineage skewing while maintaining the stem cell expansion qualities as was seen in limiting dilution studies of murine bone marrow transplantation [20]. We thus set out to evaluate the biologic effect of this transgene on human mobilized peripheral blood CD34+ cells, the graft of choice in the setting of gene therapy for genetic hematologic disease. Furthermore encouraging results would support investigation into a transient delivery of the protein product to cell culture that might facilitate HSC expansion while maintaining normal hematopoiesis and differentiation long term.

The data here suggest that the NUP98-HOXA10HD fusion gene has activity at least at the human short term repopulating hematopoietic progenitor level when tested in mobilized peripheral blood stem cells. This is evidenced by the significantly higher level of engraftment of transplanted human cells in the NSG mice in the NUP98-HOXA10HD transduction groups compared to the GFP vector control (Fig 5) as well as higher CD34+GFP+ engraftment (Fig 6). In the groups where cells were in liquid culture prior to transplant for only the time needed for transduction there was no significant difference from the untransduced Mock. However when the transduced cells were cultured for 1 week before transplant the NUP98-HOXA10HD group had significantly more engrafting cells compared to both the untransduced and GFP controls. There may be some negative effect of the transduction process itself as the GFP control appeared to have less engraftment than the Mock control making the comparison between the transduction groups of GFP control and NUP98-HOXA10HD even more important. It was also observed that in the expanded group where the mean bulk transduction by FACS analysis for GFP was 45% in the NUP98-HOXA10HD groups at day 11 post transduction, the mean GFP positivity for stably engrafting cells in the NSG mice was approximately 75%. This demonstrates a survival advantage for the NUP98-HOXA10HD containing cells compared to the untransduced ones leading to an enrichment of their numbers.

In general the myeloid/lymphoid engraftment was similar to that of untransduced controls, however it was noted in a few cases there was a tendency to more myeloid distribution in the NUP98-HOXA10HD group (Fig 7). This may not be a surprising finding given that NUP98-*HOX* fusion genes with co-operating mutations are associated with myeloid lineage leukemia[23–26]. No evidence of disturbed differentiation was seen in the experiments discussed in our report.

In recognizing the limitations of the NSG mouse transplantation model to only show short term repopulation and that serial transplantation in this mouse model has been quite challenging in our experience likely due to the low frequency of CD34+ cells that are present months after primary transplant, CFU-C assay of the engrafted cells was done to test their “stemness.” As higher number of colonies in the NUP98-HOXA10HD compared to controls was observed in the majority of experiments it further suggests an effect of the fusion gene to either maintain or expand the hematopoietic progenitor compartment (Fig 8). It also appears that the effects were donor dependent as donor 3 showed an advantage for both unexpanded and expanded groups of NUP98-HOXA10HD (Fig 8c and 8f).

In our experiments with human CD34+ cells the in vitro data did not clearly predict the in vivo expansive effect of NUP98-HOXA10HD observed. A possible explanation for this finding is given that the repopulating cells within the CD34+ fraction are rare, liquid culture conditions may not support these primitive cells as opposed to the in vivo model where engraftment may support HSCs and thus show an expansive difference there. An interesting finding was that assessment of CFU from cells in culture showed similar numbers of total colonies but lower GFP positive colonies in the NA10HD group than in the MSCVGFP group. This does raise the possibility that these cells are being kept in a less differentiated state by NA10HD and thus have less GFP colonies. If cells were being maintained in a more “stem-like” state it would be in keeping with the higher CD45+ and CD34+GFP+ engraftment of the NSG mice in the NA10HD groups seen 3 months after transplant of the CD34+ transduced cells. An important question is how durable is this expansive effect and will this hold true for long term repopulating cells as well. A competitive repopulation experiment using a non-human primate model might be useful.

The expansive effects of NUP98-HOXA10HD seen here are of particular interest to the field gene therapy for hematopoietic stem cells which are believed to decrease in number during the days of culture necessary for gene transfer to take place. The expansive effect on hematopoietic progenitors may be even more evident in a transient delivery where cells are exposed to the effect from the start of culture rather than the time delay necessary for pre-stimulation in cytokine containing medium, transduction and eventual translation of the fusion-gene to a biologically relevant level. The relatively variable transduction efficiency of lentiviral vectors as seen among different human donors would also be avoided by a uniform transient delivery method. Overall, the data suggest there may be merit in developing a NUP98-HOXA10HD transiently delivered protein for culture conditions for human CD34+ cells as we saw expansive effects despite there likely being variable response to expansion across the different donors. In diseases that have the potential to be cured by gene transfer to hematopoietic stem cells such as thalassemia and sickle cell disease, once gene modified cells have successfully engrafted and are producing all cell lineages appropriately, the continued expression of a stem cell expanding agent such as NA10HD may be unnecessary and potentially harmful. While in the short term there may not be any discernible negative effects, there likely exists a higher chance of an aging HSC that acquires a genetic mutation outgrowing as a malignant clone with ongoing expression of NA10HD. While our experimental model used continued expression of NA10HD by gene transfer to demonstrate the effect of the fusion protein, the ideal use of NA10HD would be transient delivery during HSC expansion, transduction and possible in the peri-engraftment period. Thus an optimized NA10HD protein as a cell culture additive and short term infusion for gene therapy recipients after reinfusion of gene modified HSCs would be an attractive approach. Our results also provide strong impetus to utilize NUP98-HOX10HD to enhance HSCs to search for underlying genes and pathways that may provide additional clues for novel methods to expand HSCs that would avoid gene manipulation or addition of foreign proteins to a cell culture. Investigation of additive expansive effects of NUP98-HOXA10HD on HSCs with other small molecules may also prove even more effective if different pathways are being used and should also be explored.

## Materials and Methods

### Cells

293T (adherent human embryonic kidney cells) used in lentiviral vector production were cultured in D10 medium which consisted of Dulbecco's modified Eagle's medium (CellGro, Manassas, VA), supplemented with 2 mmol/l L-glutamine (CellGro, Manassas, VA), 10% heat-inactivated fetal bovine serum (HyClone, Thermo Scientific, Logan, UT), and 50 IU/ml penicillin G/50 μg/ml streptomycin (designated hereafter as 1× P/S) (CellGro, Manassas, VA).

Mouse bone marrow cells were obtained from 8–12 week old C57BL/6 mice and lineage depleted using the autoMACS^™^ system and Lineage Cell Depletion Kit (Miltenyi Biotec, Auburn, CA). Cells were cultured in Dulbecco's modified Eagle's medium (CellGro, Manassas, VA), supplemented with 2 mmol/l L-glutamine (CellGro, Manassas, VA), 20% heat-inactivated fetal bovine serum (HyClone, Thermo Scientific, Logan, UT), 50 IU/ml penicillin G, 50 μg/ml streptomycin (CellGro, Manassas, VA), and cytokines: 20ng/ml murine IL-3, 50ng/ml human IL-6, and 50ng/ml murine SCF (all from Peprotech, Rocky Hill, NJ).

Human CD34+ cells were obtained from volunteers per a protocol approved by the St Jude Children's Research Hospital (Memphis, TN) institutional review board as well as purchased through Key Biologics, LLC (Memphis, TN). The studies were conducted in accordance with the Declaration of Helsinki. For St Jude collections volunteers gave written informed consent and were compensated for their time and expenses. The St Jude protocol used granulocyte colony stimulating factor four days before apheresis, and CD34^+^ cells were purified from the peripheral blood collection (single donor used with 88% purity) in the Human Applications Laboratory at St Jude Children's Research Hospital using the CliniMACS device (Miltenyi Biotec, Auburn, CA) according to the manufacturer's protocol. The Key Biologics, LLC purchased product was collected using a 5 day schedule of granulocyte colony stimulating factor with two days of apheresis, and CD34^+^ cells were purified from the peripheral blood collection (≥98% purity) in the Human Applications Laboratory at St Jude Children's Research Hospital also using the CliniMACS device as previously stated. For the transplantation experiments, CD34^+^ cells processed from the Human Applications Laboratory were immediately placed on ice and used without freezing.

### Plasmids and vector production

pCAG4 RTR2, pCAG-kGP1.1R, pCAG VSV-G, pCL20c MSCV GFP have been previously described [27, 28]. pCCL-c-MNDU3 FNA10 PGK GFP which encodes FLAG tagged NUP98-HOXA10HD (FNA10) was generously provided by Keith Humphries.

Viral vectors were prepared using the previously published transient HIV lentiviral system developed at St Jude Children’s Research hospital[29], collected in Stemline II medium (Sigma-Aldrich, St. Louis, MO, Cat. No. S0192) and stored in 5 ml aliquots at -80°C. Vector preparations were titered on 293T cells in a standard manner.

### Transduction of lineage negative (lin-) mouse bone marrow cells

Lineage depleted bone marrow cells from C57BL/6 mice were prestimulated in cytokine containing media for 48 hours and then transferred to RetroNectin coated 6 well plates (Takara Bio USA; cat. no. T100B) at a concentration of 5 x 10^5^/ml and incubated for four days with viral particles at a multiplicity of infection of 15 with media containing polybrene (Hexadimethrine bromide, Sigma-Aldrich, St. Louis, MO, Cat. No. H9268) at 6ug/ml. Cells were removed from the RetroNectin plates by scraping with a disposable cell lifter and maintained in culture with fresh media in 6-well plates.

Varying amounts (2 x10^3^-1x10^5^) of cultured lineage negative cells were plated for CFU-C assay weekly by mixing cells with 1 ml of methocult per plate (StemCell Technologies; cat. no. 03434 Methocult^®^ GF M3434) and plating onto 35-mm plates, and incubated at 37°C for 7–10 days and the proportion of GFP^+^ colonies was determined by fluorescent microscopy.

### Transduction and assay of CD34^+^ cells

For each donor used, equal number of CD34+ selected cells (range among donors 5.5–7 x 10^6^) were seeded on the day of processing on a ten centimeter plate that was coated with RetroNectin; 10 ml of Stemline II medium supplemented with 100 ng/ml each of recombinant human Flt3L (CellGenix, Freiburg, Germany; cat. no. 1405), SCF (CellGenix; cat. no. 1401), and TPO (CellGenix; cat. no. 1407) and gentamicin sulfate 50 mcg/ml (Lonza, Cat. No. 17-518Z), were added to each plate. Stemline medium (Sigma-Aldrich, St. Louis, MO, Cat. No. S1694) supplemented with 2 mmol/l L-glutamine was substituted for Stemline II when not available from the manufacturer. The cells were incubated at 37°C for approximately 24 hours. The cell medium was then replaced with viral preparations that were mixed with fresh cytokine containing media including 0.4 μg/ml protamine sulfate (Sigma-Aldrich, St. Louis, MO, Cat. No. P4020) and incubated at 37°C overnight. The final viral vector concentrations were 1–1.5 x 10^7^ infectious particles/ml. The cells were then removed from the RetroNectin coated plate using a combination of pipetting and 2 ml non-enzymatic cell dissociation buffer (Sigma-Aldrich, St. Louis, MO, Cat. No.C5789) applied to the cell monolayer for 5–10 minutes followed by 3 ml of phosphate-buffered saline (D-PBS, Dulbecco's Phosphate Buffered Saline, Manassas, VA, Cat. No. 21-031-CV) added and then all pipetted up.

In each experiment involving transplantation into immunodeficient mice, four identical plates were processed for each vector, two of which were used to transplant 24 hours after first exposing to viral preparations, and the other two maintained in extended culture for 1 week prior to transplant. For extended culture, cells were maintained at a concentration of 5–10 x 10^5^ cells/ml on untreated ten centimeter plates until the cells were transplanted. The proportion of GFP^+^ cells was determined by flow cytometric cell sorting of cells plated for extended culture on 6-well plates. Two hundred CD34^+^ cells were mixed with 1 ml of methocult (StemCell Technologies; cat. no. H4434 GF Methocult^®^), and plated onto 35-mm plates, incubated at 37°C for 10–14 days and the proportion of GFP^+^ colonies was determined by fluorescent microscopy.

The remainder of the CD34^+^ cells from two ten centimeter plates were collected in phosphate-buffered saline with 1x P/S supplemented with 2% heat-inactivated fetal calf serum and 300 μl containing 1 x 10^6^ starting cell equivalents were injected into the tail veins of at least ten female NOD/SCID/IL2rγ^null^ mice (NSG) that were 8–12-weeks old. The mice were injected intraperitoneally with 35 mg/kg busulfan (Busulfex; PDL BioPharma, Redwood City, CA) 24 hours before transplantation. Housing and care of the animals were in accordance with a protocol approved by the Institutional Animal Care and Use Committee at St Jude Children’s Research Hospital. Twelve to fifteen weeks post-transplantation, mice were sacrificed and bone marrow cells were harvested from tibias and femurs into D-PBS supplemented with 2% heat-inactivated fetal calf serum and 1x P/S. Samples from individual mice were prepared as single-cell suspensions and passed through a nylon cell strainer (BD Biosciences, San Jose, CA) to remove debris and then stained with the following monoclonal antibodies per the manufacturer's instructions: allophycocyanin-conjugated antihuman CD45 (Clone HI30 BD cat. no. 555485), phycoerythrin-Cy7-conjugated antihuman CD19 (Clone SJ25C1 BD cat. no. 557835), phycoerythrin-conjugated antihuman CD15 (Clone HI98 BD cat. no. 555402), and phycoerythrin-conjugated antihuman CD33 (Clone WM-54 DAKO Code No. R0745). The efficiency of gene transduction was defined by the percentage of human cells expressing GFP. The GFP-expressing hCD45^+^ population was further analyzed for either myeloid (CD33/15) or B lymphoid (CD19) subpopulations. Dead cells stained with 4′-6-diamidino-2-phenylindole were excluded from the analysis. This analysis (four-color flow cytometric analysis) was conducted using a FACS Aria (BD Biosciences). For CFU-C analysis, 4 x10^4^ sorted human CD45^+^ cells were mixed with 1 ml of H4434 GF Methocult^®^per plate, and plated onto 35-mm plates, incubated at 37°C for 10–14 days and the proportion of GFP^+^ colonies was determined by fluorescent microscopy.

The average vector copy number was determined by Realtime qPCR, using a probe set specific for the lentivirus ψ-region, and normalized to values obtained using a probe set specific for human N-RAS, as previously described[30].

All animal research was performed under approval of the St Jude Children's Research Hospital's Institutional Animal Care and Use Committee (IACUC)

### Statistical analysis

Bar graph data represent the mean +/- standard error of the mean (SEM) unless otherwise specified. Statistically significant differences in the means (*P* value <0.05) was computed using the Student’s t-test and Mann-Whitney U test (GraphPad Prism 5 and 6) as highlighted.

## Supporting Information

S1 Fig Percentage of GFP expressing cells correlates weakly with CD45+ engraftment. NSG mice assessed 12–15 week post transplantation with lentiviral transduced CD34+ cells found to have a high vs. low (>65% vs <65%) percentage of GFP+ cells in the NA10HD groups. A weak positive correlation was found between GFP+ cells and CD45+ cell engraftment (Spearman correlation coefficient, r = 0.519, p <0.001).
(TIF)
